# The Specificity and Polymorphism of the MHC Class I Prevents the Global Adaptation of HIV-1 to the Monomorphic Proteasome and TAP

**DOI:** 10.1371/journal.pone.0003525

**Published:** 2008-10-24

**Authors:** Boris Schmid, Can Keşmir, Rob J. de Boer

**Affiliations:** 1 Institute of Theoretical Biology, Utrecht University, Utrecht, the Netherlands; 2 Academic Biomedical Centre, Utrecht University, Utrecht, the Netherlands; University of California San Francisco, United States of America

## Abstract

The large diversity in MHC class I molecules in a population lowers the chance that a virus infects a host to which it is pre-adapted to escape the MHC binding of CTL epitopes. However, viruses can also lose CTL epitopes by escaping the monomorphic antigen processing components of the pathway (proteasome and TAP) that create the epitope precursors. If viruses were to accumulate escape mutations affecting these monomorphic components, they would become pre-adapted to all hosts regardless of the MHC polymorphism. To assess whether viruses exploit this apparent vulnerability, we study the evolution of HIV-1 with bioinformatic tools that allow us to predict CTL epitopes, and quantify the frequency and accumulation of antigen processing escapes. We found that within hosts, proteasome and TAP escape mutations occur frequently. However, on the population level these escapes do not accumulate: the total number of predicted epitopes and epitope precursors in HIV-1 clade B has remained relatively constant over the last 30 years. We argue that this lack of adaptation can be explained by the combined effect of the MHC polymorphism and the high specificity of individual MHC molecules. Because of these two properties, only a subset of the epitope precursors in a host are potential epitopes, and that subset differs between hosts. We estimate that upon transmission of a virus to a new host 39%–66% of the mutations that caused epitope precursor escapes are released from immune selection pressure.

## Introduction

Antigen presentation allows CD8^+^ T cells to monitor the protein content of a cell and detect the presence of intracellular viruses [1]. The classical antigen presentation pathway consists of three main steps: the (immuno-)proteasome, which cleaves cytoplasmic proteins into peptide fragments; the transporter associated with antigen processing (TAP), which transports peptide fragments into the endoplasmic reticulum; and the major histocompatibility complex (MHC) class I, which binds a small fraction of these endoplasmic peptide fragments [2], and transports them to the cell surface [3]–[5]. The peptide fragments that are processed by the proteasome and transported by TAP are commonly called ‘epitope precursors’.

Of these three steps in the antigen presentation pathway it is only the MHC that is highly polymorphic, which is thought to have evolved because of the *rare allele advantage* [6]–[8]: hosts that carry rare MHC alleles are less likely to be infected by viruses that are adapted to escape the host's MHC alleles than hosts with common MHC alleles, because it is less likely that these viruses come from a host with the same rare MHC alleles. Therefore hosts with rare MHC alleles are thought to have a fitness advantage. Indeed, hosts that were infected with preadapted variants of the human immunodeficiency virus 1 (HIV-1) were found to progress rapidly to AIDS [9]–[11]. However, if viruses adapt to escape the epitope precursors [12]–[15], which are created by the monomorphic proteasome and TAP, the protective effect of the MHC polymorphism and the fitness advantage of hosts with rare MHC alleles would be lost.

We studied the ability of HIV to generate and accumulate epitope and epitope precursor escapes, using algorithms that can reliably predict the likelihood of proteasomal cleavage, TAP transport, and MHC binding of amino acid sequences (see Material & Methods). We discovered that there is no accumulation of epitope precursor escapes on the population level: the total number of epitope precursors (as well as that of epitopes) has remained relatively constant over the last 30 years. We explored several possible causes for this lack of adaptation to the antigen processing machinery, and postulate a mechanism by which the specificity and polymorphism of the MHC prevents the adaptation of viruses to the monomorphic parts of the antigen presentation pathway.

## Materials and Methods

### CTL epitope predictions

Currently, a wide variety of algorithms [16]–[19] are available to predict MHC-peptide binding. The capacity of these algorithms to identify new epitopes has routinely been tested on experimental data [20], [21], and their accuracy has increased over time to such an extent that the correlation between predicted and measured binding affinity is as good as the correlation between measurements from different laboratories [20]. A further increase in accuracy of identifying Cytotoxic T lymphocytes (CTL) epitopes is achieved by combining the MHC binding predictors with predictors trained to mimic the specificity of the proteasome and TAP, thus creating a model of the complete antigen presentation pathway [19], [22], [23]. These pathway models come in two types: those that sum the scores of the independent steps of the antigen processing pathway, and use a threshold on the summed score (e.g. MHC-pathway [23] and NetCTL [22]), and those that eliminate epitope candidates at each step (e.g. EpiJen [19], MAPPP [24] and the alternative implementation of MHC-pathway [23]).

**Figure 1:**
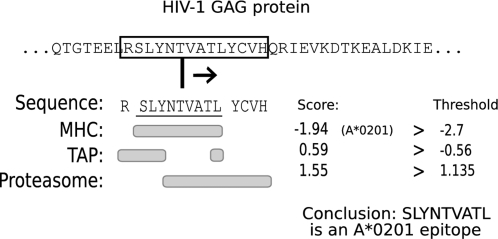
Schematic of the MHC-pathway model. A window of 14 amino acids is slided across a protein. Each of these ‘14mers’ consists of a N-terminal flanking region of 1 amino acid, a 9mer epitope candidate and a C-terminal flanking region of 4 amino acids. Beneath the 14mer the parts of the peptide that are used by the MHC, TAP or proteasome predictors are marked. Applying the 14mer to the MHC, TAP and proteasome predictors results in three different scores. If each of these scores is higher than a fixed threshold, then the 9mer embedded in the 14mer is predicted to be a CTL epitope for the MHC allele tested (in this case A*0201). If a 14mer passes at least the proteasome and TAP predictors then the 9mer embedded in the 14mer is predicted to be an epitope precursor. In the analysis of longitudinal within-host data sets, CTL epitopes are scored as escapes if mutations in the 14-mer lower one of the three scores below the corresponding threshold.

In this study we use the alternative implementation of the MHC-pathway model [23]. We screen all possible peptide fragments of 14 amino acids within a particular protein, and eliminate those fragments that cannot be correctly processed by either the proteasome, TAP or the MHC class I molecules (Fig. 1). This approach allows us to distinguish between adaptation of a virus to antigen processing and adaptation to MHC class I binding. The threshold values for the proteasome and TAP predictors (Fig. 1) were derived by applying the MHC-pathway model to a large bacterial protein data set and selecting threshold values which correspond to the estimated specificity of the proteasome (33%) and TAP (76%) [25]. For the MHC-binding predictions we used the default threshold of −2.7, which corresponds to an IC50 threshold of 500 nM [2], [20]. As a result of using 500 nM as the threshold for MHC binding our analysis focuses on the medium to strong HIV-1 epitopes, and disregards the weaker CTL epitopes in the 500–5000 nM range in favor of a higher specificity (i.e. less false positives) of the MHC-pathway model. The dependency of our results on the selected thresholds and the selected predictor was tested by repeating the population and ancestor analysis for the HIV-1 clade B ENV, GAG and NEF proteins, using a more relaxed MHC binding criteria (5000 nM), as well as using another prediction algorithm, NetCTL [22]. The predictors used in this paper are available through a web interface (http://tools.immuneepitope.org/analyze/html/MHC_binding.html 2006-01-01 version). Note that we excluded 2 of the 34 available MHC predictors. The A*3002 predictor was very non-specific at our thresholds, predicting MHC binding in as much as 9944 out of 50.000 HIV-1 derived 9mers (20%). The B*0801 MHC predictor appeared to be very specific, and predicted no MHC binding in 50.000 HIV-1 derived 9mers at the thresholds we use.

### Prediction quality

Epitope predictors are routinely tested on large sets of epitopes derived from various pathogens [20], [26]. More recently, Larsen et al. [21] tested the performance of four widely used predictors on a data set of only HIV-1 epitopes. In that study, NetCTL and MHC-pathway came out as the best performing algorithms. MHC-pathway is estimated to recover 80% of the known epitopes at a specificity of 90%, and recover 30% of the known epitopes at a specificity of 99.3%. However, Larsen et al. [21] stressed that these specificity ratings were underestimates, as the test data set was build with the assumption that any peptide that isn't a known CTL epitope must be a non-epitope, due to the lack of confirmed non-epitopes. As a result, many of the correctly predicted but experimentally not yet verified epitopes were scored as erroneous predictions. A second issue that makes the exact estimation of prediction quality difficult is that many of the experimentally confirmed epitopes are based on CTL responses measured against overlapping peptide pools, and are often defined as the best responding amino acid substring within a peptide that elicits a T cell response, regardless of whether or not this substring is a peptide that can be naturally processed. A more reliable way to estimate the specificity of the predictors is to predict a set of CTL epitopes and subsequently verify CD8^+^ T cell responses against these epitopes experimentally. Schellens et al. [27] identified 18 new CTL epitopes out of a set of 22 predicted CTL epitopes in this manner (using NetCTL). This suggests that the specificity of the predictors is far higher than the benchmark estimates, and places the amount of false positive predictions at 20%. Pérez et al [28] identified 114 out of 184 predicted epitopes (38% false positives) in a similar manner, but predicted CTL epitopes for the MHC supertypes rather than genotypes, which may explain their higher rate of false positives. A more direct approach to measuring predictor quality is the use of mild acid elution and mass spectrometry to determine MHC-binding peptides. Using these techniques Fortier et al. [29] estimated the false positive rate of the MHC-binding predictors of the MHC-pathway model to be less than 2%.

### HIV-1 longitudinal within-host data

In December 2007, we performed an exhaustive search on the HIV Sequence Database (http://hiv.lanl.gov) for longitudinal within-host sequences from 4 digit HLA-genotyped patients that had not received antiretroviral therapy, and for which at least 3 matching MHC predictors were available. This resulted in a data set of 13 patients for which GAG, NEF and POL protein sequences were available (see Table S1 and S2 for sampling dates and accession numbers). All patients were infected with HIV-1 clade B, and their sample HIV-1 sequences spanned a time period of at least two years. The time between infection with HIV-1 and extraction of the early sequence sample was in all cases less than a year. Surprisingly, 8 out of 13 patients carried HLA-B5701 and/or HLA-B2705, two rare and protective alleles [30], [31], which probably reflects an observation bias in the data base. All longitudinal within-host sequences were translated from nucleotide to protein sequences with the GeneCutter tool (http://www.hiv.lanl.gov/content/sequence/HIV/HIVTools.html). For patient PIC1362(1052829) multiple sequences per protein per timepoint were available, with small differences between each sequence. Not knowing which of the early timepoints (if any) was the ancestral sequence of the late timepoints complicated some of the within-host analysis. We took a prudent approach by excluding from the analysis the amino acid positions and CTL epitopes for which population dynamics effects could not be ruled out. For example, if a particular epitope was present in the majority of the early timepoint sequences, but not in any of the late timepoint sequences, there are two possibilities: the early timepoint sequences that still contained the epitope escaped it, or the early timepoint sequences that did not contain the epitope outcompeted those sequences that did contain the epitope. In such cases the epitope was excluded from the analysis.

### HIV-1 population data

**Figure 2:**
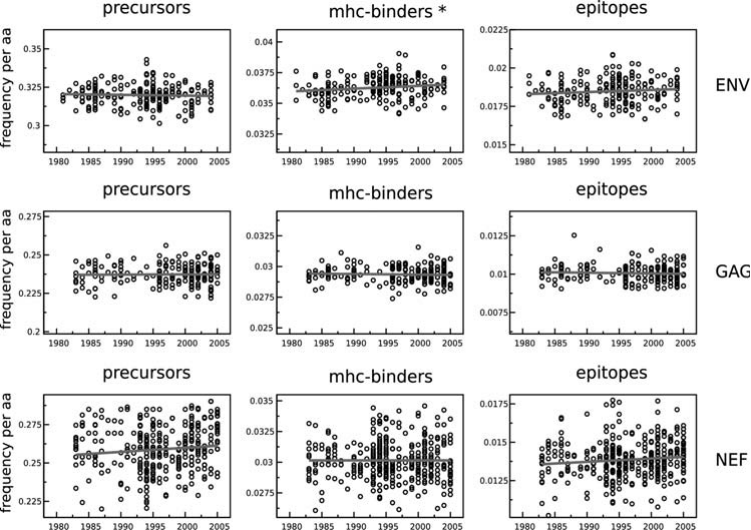
The predicted density of epitope precursors, MHC-binding peptides and CTL epitopes in ENV, GAG and NEF stay constant over time. The density (expressed as frequency per amino acid) is plotted on the y-axis with the same scale factor within each column, which makes it possible to compare differences between proteins. The densities for MHC-binding and CTL epitopes are averaged over the 32 MHC-binding predictors. *: significant increase over time (Kendall Tau rank correlation test, p<0.01).

**Figure 3:**
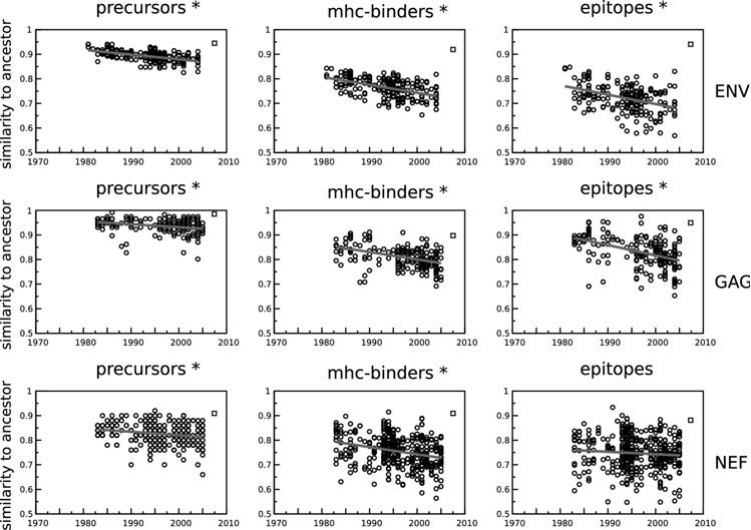
The predicted fraction of ancestral epitope precursors, MHC-binding peptides and CTL epitopes present in the HIV-1 clade B sequences declines over time. The predicted fraction is plotted per sequence as a dot on the y-axis. Ancestral epitope precursors are defined by their C-terminal position (Fig. 1, 10th amino acid from the left) in the aligned sequences [3]. Similarly, MHC-binding peptides and CTL epitopes are defined by their C-terminal, but also by the MHC that they are predicted to bind to. *: significant decrease over time (Kendall Tau rank correlation test, p<0.01). The consensus sequence [65] is plotted as a square on the right-hand side of each panel.

The HIV-1 population data set used in this paper is the HIV-1 clade B subset of the aligned HIV-1 Sequence Compendium 2002 (Dec 2007 version) [32]. This data set was pruned of sequences for which the sampling date was unknown. The sequence compendium consists of 9 aligned fasta files, one for each of HIV-1's proteins. The number of available HIV-1 clade B sequences in the compendium differs per protein and ranges from 96 to 386 sequences (see Table S3 for details). The correlations in Fig. 2 and Fig. 3 were determined with the Kendall Tau rank correlation test [33] of the statistical package R [34]. The predicted HIV-1 clade B ancestor sequence [35] (available at http://www.hiv.lanl.gov/content/sequence/NEWALIGN/align.html) was aligned to the population data set with HMMER 2.3.2 [36], a profile hidden Markov model.

## Results

### Adaptation to the human population

To determine whether HIV has exploited the lack of polymorphism of the proteasome and TAP, we predicted the number of epitope precursors in a HIV-1 clade B sequence population data set with samples from 1980 to 2005 (see Material & Methods for details on the HIV-1 Sequence Compendium data set [32] and the quality of the MHC-pathway model [21]). We plotted the predicted epitope precursor density of each HIV-1 sequence against its sampling date to study the changes over time (Fig. 2, first column & Table S3). Using 32 MHC peptide binding predictors, the same procedure was followed to plot the average density of MHC-binding peptides, and the average density of CTL epitopes. In all three cases there was no sign of any large-scale adaptation of HIV-1 clade B to its human host over the last 30 years: the number of epitope precursors, MHC-binding peptides and CTL epitopes per HIV-1 sequence remained constant over time. Differences existed mainly between proteins: the envelope protein (ENV) seemed more immunogenic and had a higher density of precursors, MHC-binders and CTL epitopes than the other proteins, and the NEF protein showed a far greater variability between sequences than the other proteins. In addition to the proteins shown in Fig. 2, the same analysis was performed for the other proteins of HIV-1 (Table S3), for two other HIV-1 clades (clade C (Table S4) and clade A1 (Table S5)), as well as for human subpopulations (Kroatia, UK & USA (Table S6)) within the HIV-1 clade B population data set. This resulted in a total of 102 tests, of which 14 were found to be significant at a p-value of <0.01 (Kendall Tau rank correlation test). However, in 6 out of these 14 significant cases HIV-1 was gaining epitope precursors, MHC-binding peptides, or CTL epitopes over time. The 7 cases in which the density significantly decreased over time were not consistently occurring in the same proteins when comparing different HIV-1 clades, nor were they consistently affecting the same step in the antigen presentation pathway. This makes it unlikely that these 7 correlations reflect the adaptation of HIV-1 to its human host. We verified our results by repeating the analysis for ENV, GAG and NEF using a more relaxed MHC binding threshold (5000 nM), as well as with a different prediction algorithm [21] (data not shown). This did not result in a qualitative difference, except that according to the NetCTL predictions [21] the epitope precursor density in HIV-1 NEF decreased slowly, but significantly over time (Kendall Tau rank correlation test, p<0.01. Epitope precursor density is predicted to change from 49 precursors in 2008, to an estimated 46 precursors in 2032).

That the number of MHC-binding peptides in HIV-1 remained constant over time (Fig. 2, middle column) was to be expected, based on the theory that the MHC polymorphism prevents pathogens like HIV-1 from escaping MHC binding on a population level [8], [37], and earlier reports that the virulence of HIV-1 had not changed over time [38], [39]. However, other studies reported that HIV-1 was capable of adapting to common MHC alleles [40]–[42], and suggested that HIV-1 was adapting to its new human host population. Follow-up studies on Moore et al. [40] showed that their analysis was sensitive to founder effects in the viral lineage [43] and that without these effects the adaptation of HIV-1 to the population could only be detected for a small number of amino acids [44].

In line with our a priori expectations, Yusim et al. [45] proposed that the clustering of epitopes in HIV-1 proteins was a result of adaptation of the virus to the monomorphic proteasome and TAP. However, our results (Fig. 2) refute that expectation: HIV-1 has not accumulated epitope precursor escapes over the last 30 years. In this paper we explore possible reasons for the apparent lack of adaptation of HIV-1 to the monomorphic antigen processing machinery.

### Selection pressure by CD8^+^ T cells

A simple explanation for why we find that HIV-1 is not accumulating CTL epitope and epitope precursor escapes (Fig. 2) would be that CD8^+^ T cells exert too little selection pressure on the virus. The role of CD8^+^ T cells in controlling a chronic HIV-1 infection has been under debate [46], [47], but the strongest evidence that the virus is under selection pressure of CD8^+^ T cells is that certain immune escape mutations are rapidly reverted to the wildtype upon entering an HLA-mismatched host [10], [48]–[50]. Additional evidence comes from CD8^+^ T cell depletion studies of chronic SIV infections in monkeys [51]–[53], from studies that show that MHC-heterozygous hosts progress slower to AIDS than homozygous hosts [54], and from correlates between HIV-1 disease progression and the presence or absence of certain MHC class I molecules [30], [31].

In addition to the strong evidence on the selection pressure imposed by CD8^+^ T cells from the current literature, we studied the CD8^+^ T-cell mediated immune selection pressure on HIV-1 by testing whether amino acid replacement mutations happen preferentially in CTL epitopes and their flanking regions. For this purpose we data-mined the Los Alamos HIV database for longitudinal within-host HIV-1 sequence data from MHC genotyped and treatment-naive patients (see Material & Methods, and Supporting Information). This resulted in 13 patients for which GAG, NEF and POL protein sequences were available (see Table S1, S2). We compared for each of these proteins the number of amino acid replacements that occurred within predicted epitopes or their flanking regions to the expected number of mutations. This expected number of mutations is based on the fraction of the protein that the epitopes and their flanking regions covered (‘epitope cover’). We found a trend towards mutations occurring within CTL epitopes or their flanking regions for the three HIV-1 proteins that were tested (Wilcoxon signed rank test: p = 0.09, with 15 out of 21 samples following the trend).

A surprising observation based on our immune selection pressure study was that the predicted CTL epitopes within a single host can cover a large fraction of the viral proteome. For those samples in the longitudinal within-host data set where predictors were available for all four of the Human Leukocyte Antigen A (HLA-A) and HLA-B alleles of the host, the epitope cover ranged from 12% to 74%. The average epitope cover of a single MHC allele for the HIV-1 clade B HXB2 reference sequence (accession: K03455) was 17%, and all 32 MHC predictors together covered 94% of the HIV-1 HXB2 virus proteome.

That we find no significant correlation between the location of mutations and predicted CTL epitopes might be due to differences between CTL epitopes in the strength of the immune selection pressure imposed on them, which would reduce the detection power of our method of testing for selection pressure. Yewdell et al. [55] indicated that only half of all CTL epitopes can trigger a CD8^+^ T cell response. The underlying mechanism is poorly understood, but possibly involves self-tolerance [56], [57]. Zafiropoulos et al. [58] and Frater et al. [50] showed that the selection pressure imposed on the virus differs between CTL epitopes. A possible cause for this variation is whether an epitope is presented early or late during the infection of a cell [59]–[61]. Since the trend we find is confirmed by the current literature, we assert that the lack of predicted adaptation in HIv-1 (Fig. 2) is not likely to be due to a lack of immune selection pressure.

### Short timespan of population data set

Another possible reason why we find that HIV-1 is not accumulating CTL epitope and epitope precursor escapes (Fig. 2) would be that the timespan of our population data set (30 years - from 1976 to 2006) is too short to detect an evolutionary process like the adaptation of a virus to its host. To test this, we predicted the epitope precursors of the putative HIV-1 clade B ancestor sequence [35] and plotted the fraction of ancestral epitope precursors contained in each sequence of our population data set against the sampling date (Fig. 3). In this way the ‘immunological similarity’ of a sequence with the ancestor sequence can be visualized. This similarity is expected to decline over time, based on the destruction of ancestral epitope precursors by neutral amino acid substitutions [62]–[64], and by the accumulation of escapes from CD8^+^ T cell responses within hosts. If the time covered by our population data set is sufficient, we should see a decrease over time in the immunological similarity of current-day sequences to the ancestral HIV-1 sequence.

Indeed, for ancestral epitope precursors as well as for ancestral MHC-binding peptides and CTL epitopes, we found that the density declined significantly over time in the six largest proteins of HIV-1 (Kendall Tau rank correlation test, p<0.01 in 17/18 tests). The only exception was a non-significant decrease in the number of predicted ancestral epitopes in HIV-1 NEF (Fig. 3, bottom right panel). Analysis of the NEF protein subset of the population data set revealed that in the Kroatian population the number of ancestral epitopes in NEF was increasing over time. Whether this increase reflects a particular adaptation of the virus, or is due to a founder effect in the Kroatian subpopulation that was oversampled in the HIV Sequence Compendium data set is not known. The three smallest proteins of HIV-1 (TAT, VPR and VPU) yielded no significant results, which indicates that at protein sizes of less than 100 amino acids our method becomes insensitive.

The HIV-1 clade B consensus sequence (Fig. 3, open square on the right-hand side of each panel) is more similar to the predicted ancestral sequence than most of the HIV-1 sample sequences themselves are, which indicates that HIV-1 is undergoing divergent evolution. This is inconsistent with the idea that HIV-1 is undergoing a large-scale global adaptation to the human host (which would imply a convergent evolution process). Based on the results presented thus far, we conclude that the evolution of HIV-1 seems largely determined by the loss of ancestral epitopes due to antigenic drift, by local adaptation of the virus to each individual host, and by the reversion of earlier adaptations.

Finally, the rate at which ancestral epitopes and epitope precursors disappear (Fig. 3, grey lines) gives a novel way to estimate in what year HIV-1 clade B was introduced into the human population. The age of the ancestral HIV-1 B sequence can be predicted by extrapolating the regression line back to where the fraction of ancestral epitopes or epitope precursors in HIV-1 sequences becomes one, assuming that the loss over time has been linear. Each protein and each category (precursor, MHC binding, epitopes) generates a separate prediction for the age of the ancestral sequence. For the larger genes ENV, GAG and POL, the estimated ancestral age is 1939±13, whereas for the smaller genes it is 1900±54 years. The estimate for the larger genes concurs with the findings of Korber et al. [35], who dated the ancestral sequence on 1920–1940.

Summarizing: the analysis of the loss of ancestral epitope precursors shows that 30 years is long enough to pick up evolutionary processes in the larger proteins of HIV-1 (>100 amino acids). Therefore, the lack of adaptation to epitope precursors in HIv-1 (Fig. 2) should not be attributed to the relatively short time span of the population data set.

### Rarity of precursor escapes

Brander et al. [66] hypothesized that the proteasome and TAP should be rather non-specific for their substrate in order to fulfil their intracellular functions. Therefore, most mutations should not affect antigen processing, and as a result epitope precursor escapes would be harder to generate than MHC binding escapes. Although several studies have clearly shown that antigen processing escapes do exist [64], [66], the frequency of successful antigen processing escapes in vivo could be so low that these kind of escapes play no role in the evolution of HIV-1, which would explain why HIV-1 is not accumulating epitope precursor escapes (Fig. 2).

We used the MHC-pathway model to determine the frequency of antigen processing escapes in a longitudinal within-host HIV-1 sequence data set (27 HIV-1 proteins from a total of 13 different patients, see Material & Methods, Table S1 and S2). We found that 38 out of a total of 375 predicted CTL epitopes were escaped by the virus (10.1%) during the time spanned by the longitudinal within-host data set. Of these 38 escaped CTL epitopes, 34 (89%) contained one or more mutations that prevented the peptide from binding to its associated MHC molecule, and 6 (16%) contained one or more mutations in the epitope or the epitope's flanking region that prevented antigen processing of the epitope precursor.

A second way to study the frequency of epitope precursor escapes is to study the predicted effect of a single amino acid substitution on the number of CTL epitopes in a HIV-1 protein. While this approach completely ignores functional constraints on proteins, it has the advantage that we can calculate the average effect of a single mutation on the escape of CTL epitopes. This procedure was repeated a large number of times, until on average each amino acid in the HXB2 reference sequence had been mutated five times (previous mutations were reversed before a new one was generated). In 3.8% of the cases this procedure resulted in the loss of one or more epitopes per MHC allele. In 38% of these escape mutations the epitope precursor was no longer processed correctly by the proteasome or TAP (Table 1).

**Table 1:** Effect of random amino acid substitutions on the average loss and gain of CTL epitopes per MHC allele in the HIV-1 HXB2 reference sequence.

	loss of epitopes	gain of epitopes
**Mutations affecting epitope count**	3.8%	4.3%
due to processing escapes	38%	40%
due to MHC-binding escapes	80%	81%

The amino acid substitution simulations on the HIV-1 HXB2 reference sequence showed that new CTL epitopes are predicted to be readily created by random mutations (Table 1). Similarly, comparing the early and late timepoints of the longitudinal within-host data set showed that 56 new CTL epitopes were predicted to have arisen. Although the generation of new CTL epitopes during within-host evolution seems counter-intuitive, this has been shown to occur in reality [67], [68]. There are several reasons why new CTL epitopes could come about, despite immune selection pressure: 1) a single mutation could escape an epitope against which a strong immune response was directed, while at the same time create a new epitope with a weaker response [67], 2) if a small number of strong immune responses determine most of the fitness of the virus, adding a single weak response to the existing weak responses has a negligible effect on the fitness of the virus, 3) the new epitopes might not be recognized by any of the CD8^+^ T cell receptors of the host [55], and 4) the time between the generation of a new epitope and the expansion of a CD8^+^ T cell response against this new epitope provides a time window during which new CTL epitopes in a HIV-1 sequence are not penalized [69].

The analysis of the longitudinal within-host data set and the simulated HIV-1 HXB2 reference sequence mutations established that antigen processing escapes occur relatively frequently, and thus that the predicted lack of antigen processing adaptation of HIV-1 (Fig. 2) is not because precursor escapes are too hard to generate. The analysis also showed that new CTL epitopes are frequently generated during the within-host evolution of the virus.

### Polymorphism and Specificity

**Figure 4:**
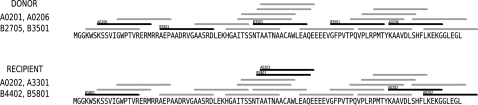
The effect of MHC specificity and polymorphism on the selection pressure on epitope precursors. This example shows the first 100 amino acids of the NEF protein of the HIV-1 HXB2 reference sequence (accession: K03455), and the precursors and epitopes for two fictitious hosts. The lines represent the epitope precursors generated by the monomorphic proteasome and TAP. The black lines depict those epitope precursors that are used by the MHC alleles. In this example, 4 out of 5 epitope precursors were released from selection pressure, as were 32 out of 70 amino acids.

In the previous sections we investigated three possible explanations for the predicted lack of adaptation of HIV-1 to the monomorphic antigen processing pathway (Fig. 2), but found no compelling evidence for any of them. Here we propose an alternative explanation: as each MHC class I allele utilizes only a small fraction of the available HIV-1 epitope precursors, not all of the epitope precursors in a host are under selection pressure. When a virus is transmitted from one host to a new host with a different set of MHC molecules a large number of the epitope precursors that were previously under immune selection pressure are no longer so. Escape mutations in those epitope precursors can subsequently revert to the wildtype sequence. A visual example of this mechanism is depicted in Fig. 4, in which a HIV-1 protein is passed from one fictitious host to another.

**Figure 5:**
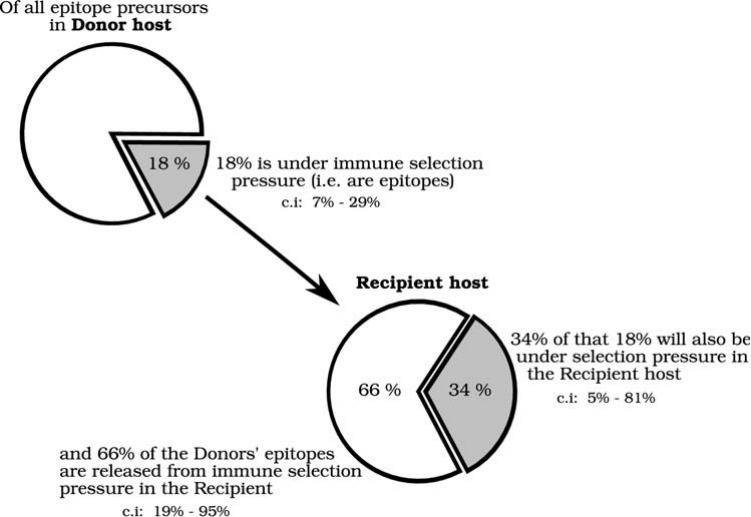
The number of epitope precursors that bind to MHC alleles in the donor host but no longer in the recipient host is calculated based on a 1000 simulated passages of the HIV-1 HXB2 reference sequence (accession: K03455) between two hosts. The hosts are randomly created from a set of 18 different HLA-A molecules and 14 different HLA-B molecules. On average, the 4 MHC alleles of a host bind 18% (145) of the 818 predicted epitope precursors. Of these 145 epitope precursors, an average of 34% can bind to one of the MHC alleles of a recipient host, whereas 66% is no longer under selection pressure. 95% confidence intervals (c.i) are shown in the figure. The overlap in epitope precursors used by the donor and recipient is partially due to overlapping MHC molecules (in 42% of the cases the randomly created donor and recipient shared by chance one or more MHC alleles), and partially due to the promiscuity of MHC alleles.

While the proposed mechanism is straightforward and plausible, its protective effect depends on the fraction of epitope precursors that is under selection pressure in the donor host, but no longer in the recipient host. This is directly influenced by the specificity and promiscuity of the MHC alleles of both host and donor: the more specific the MHC binding is, the smaller the subset of epitope precursors is that is used by the MHCs of the host, and therefore the larger the typical fraction of epitope precursors is that is released from selection pressure when the virus changes from one host to the other. We estimated this fraction with a simple model in which we create fictitious hosts with random sets of MHC alleles, and transmit the HIV-1 HXB2 reference sequence from one host to another. Each time the virus is transmitted, we calculate the fraction of the epitope precursors that were used by the MHC alleles of the donor host, but are not utilized in the recipient host. In this way we estimated that on average 18% of the epitope precursors are under selection pressure in a host, i.e. are an actual epitope in that host. 66% of these actual epitopes will be released from selection pressure in the next host (Fig. 5). Alternatively, the protective effect can also be calculated at the level of amino acid positions rather than at the level of epitope precursors. By doing so we predicted that on average 49% of the amino acid positions are under selection pressure in a random host, and that 39% of this group of 49% is released from selection pressure upon transfer of the virus to a new host. These two estimates represent the extreme ends of how much escape mutations in one epitope precursor influence the processing and presentation of another epitope precursor. The true fraction of epitope precursors that is released from selection pressure when a virus travels from one host to the next should lie somewhere in between this range of 39%–66%. Note that the range depends on the thresholds we used for proteasome, TAP and MHC binding. Increasing the specificity of the MHC binding from an IC50 of 500 nM to 50 nM increases the fraction of released epitopes to a range of 76%–83%, and lowers the average number of predicted CTL epitopes from 145 to 18 epitopes per host per viral sequence. Decreasing the specificity of the MHC binding to 5000 nM decreases the fraction of released CTL epitopes to a range of 6%–31%, but with this threshold we predict an unrealistically large number of CTL epitopes (514) per viral sequence.

Based on these predictions, we argue that the magnitude of this MHC specificity and polymorphism-dependent release mechanism is large enough to play an essential role in slowing down the adaptation of HIV-1 to the proteasome and TAP. Its exact effect on the evolution of HIV-1 will also depend on other characteristics of the virus, such as the transmission rate of the virus, and the balance between the rate of escape mutations and the rate of escape reversion in the virus [42]. Combined with our proposed mechanism, these factors will determine the eventual degree of adaptation to the antigen presentation pathway that viruses like HIV-1 can reach.

## Discussion

The total number of predicted epitope precursors and CTL epitopes in a large population data set of HIV-1 clade B sequences is not decreasing over time (Fig. 2). This is in contrast to our initial expectation that HIV-1 would be able to adapt to the monomorphic steps of the antigen presentation pathway (i.e. the proteasome and TAP) to evade the presentation of CTL epitopes derived from its proteins on the cell surface. We investigated three possible factors that could explain why we did not detect adaptation: 1) possible lack of CD8^+^ T cell selection pressure, 2) the evolutionary short timespan of 30 years of our population data set (Fig. 3), and 3) the possible rarity of epitope precursor escapes (Table 1), but found no compelling evidence for any of them.

In the last section of the results we added and discussed a fourth possibility, namely that the adaptation of HIV-1 to epitope precursors is limited by frequent loss of the immune selection pressure on epitope precursor escapes as the virus passes from one host to another (Fig. 4). We proceeded by quantifying that a typical proteasome or TAP escape mutation is released from selection in 39% to 66% of the human hosts. We propose that this loss of selection pressure on epitope precursors is one of the main factors that determine the eventual degree of adaptation to epitope precursors that HIV-1 can reach on the population level (Fig. 5). Other factors are the transmission rate, the rate at which epitope precursor escapes are acquired, and the rate at which they are lost or reverted [42].

Based on our understanding of this mechanism, we speculate that only one of the steps in the antigen presentation pathway has to be polymorphic to prevent pathogens from adapting to any step in the pathway. The mechanism functions best when the polymorphy occurs at the most specific step in the pathway, as that increases the fraction of epitope precursors that is not under selection pressure. While in humans it is the MHC class I molecules that are highly polymorphic and specific, other solutions do appear to exist. The TAP molecules of rats are more specific than the human TAP, and have a limited functional polymorphism [70], and the TAP and MHC genes of chickens are equally polymorphic on the nucleotide level [71]. We are currently exploring the conditions that determine which of the steps of the antigen presentation pathway become polymorphic using an agent-based host-pathogen model.

The lack of any large-scale adaptation of HIV-1 to reduce its number of CTL epitopes -as reported by this study- is not necessarily in contradiction with the possible fixation of certain CTL epitope escape mutations at the population level [41], [44], especially if these occur in combination with compensatory mutations [72]. However, our analysis indicates that the fixation of CTL epitope escape mutations are not occurring at a scale that makes it detectable amidst the constant destruction and generation of CTL epitopes due to neutral amino acid substitutions (Fig. 3, Table 1). Furthermore, we have only studied the adaptation of HIV-1 to escape antigen presentation by means of amino acid substitutions. HIV-1 also influences epitope presentation by blocking TAP transport [73] and downregulating MHC molecules [74]. Adaptation to the pathway could be occurring at this level, rather than at the level of individual CTL epitopes. However, current findings that individual escape mutations can have a large impact on viral load during within-host evolution [68], [75], suggests that there is still a strong selection pressure on individual CTL epitopes.

In summary: the monomorphic parts of the antigen presentation pathway are protected from viral immune escape adaptations because only a subset of the epitope precursors can be presented by the MHC alleles of a particular host. Because of the MHC polymorphism, this subset differs between hosts. As a result, epitope precursor escape mutations are frequently released from immune selection pressure when pathogens spread through a population, and can revert to the wildtype sequence. The protective effect of this mechanism increases with the polymorphism of the MHC class I and the specificity of individual MHC class I alleles.

## Supporting Information

Table S1 (0.03 MB DOC)

Table S2 (0.05 MB DOC)

Table S3 (0.05 MB DOC)

Table S4 (0.05 MB DOC)

Table S5 (0.05 MB DOC)

Table S6 (0.05 MB DOC)

## References

[1] Paulsson KM (2004). Evolutionary and functional perspectives of the major histocompatibility complex class I antigen-processing machinery.. Cell Mol Life Sci.

[2] Assarsson E, Sidney J, Oseroff C, Pasquetto V, Bui HH (2007). A quantitative analysis of the variables affecting the repertoire of T cell specificities recognized after vaccinia virus infection.. J Immunol.

[3] Craiu A, Akopian T, Goldberg A, Rock KL (1997). Two distinct proteolytic processes in the generation of a major histocompatibility complex class I-presented peptide.. Proc Natl Acad Sci U S A.

[4] Rock KL, York IA, Saric T, Goldberg AL (2002). Protein degradation and the generation of MHC class I-presented peptides.. Adv Immunol.

[5] Groothuis TAM, Griekspoor AC, Neijssen JJ, Herberts CA, Neefjes JJ (2005). MHC class I alleles and their exploration of the antigen-processing machinery.. Immunol Rev.

[6] Snell GD (1968). The H-2 locus of the mouse: observations and speculations concerning its comparative genetics and its polymorphism.. Folia Biol (Praha).

[7] Bodmer WF (1972). Evolutionary significance of the HL-A system.. Nature.

[8] Borghans JAM, Beltman JB, de Boer RJ (2004). MHC polymorphism under host-pathogen coevolution.. Immunogenetics.

[9] Goulder PJ, Brander C, Tang Y, Tremblay C, Colbert RA (2001). Evolution and transmission of stable CTL escape mutations in HIV infection.. Nature.

[10] Leslie AJ, Pfafferott KJ, Chetty P, Draenert R, Addo MM (2004). HIV evolution: CTL escape mutation and reversion after transmission.. Nat Med.

[11] Chopera DR, Woodman Z, Mlisana K, Mlotshwa M, Martin DP (2008). Transmission of HIV-1 CTL escape variants provides HLA-mismatched recipients with a survival advantage.. PLoS Pathog.

[12] Bergmann CC, Tong L, Cua R, Sensintaffar J, Stohlman S (1994). Differential effects of flanking residues on presentation of epitopes from chimeric peptides.. J Virol.

[13] Beekman NJ, van Veelen PA, van Hall T, Neisig A, Sijts A (2000). Abrogation of CTL epitope processing by single amino acid substitution flanking the C-terminal proteasome cleavage site.. J Immunol.

[14] Allen TM, Altfeld M, Yu XG, O'Sullivan KM, Lichterfeld M (2004). Selection, transmission, and reversion of an antigen-processing cytotoxic T-lymphocyte escape mutation in human immunodeficiency virus type 1 infection.. J Virol.

[15] Milicic A, Price DA, Zimbwa P, Booth BL, Brown HL (2005). CD8+ T cell epitope-flanking mutations disrupt proteasomal processing of HIV-1 Nef.. J Immunol.

[16] Peters B, Sette A (2005). Generating quantitative models describing the sequence specificity of biological processes with the stabilized matrix method.. BMC Bioinformatics.

[17] Nielsen M, Lundegaard C, Worning P, Hvid CS, Lamberth K (2004). Improved prediction of MHC class I and class II epitopes using a novel Gibbs sampling approach.. Bioinformatics.

[18] Parker KC, Bednarek MA, Coligan JE (1994). Scheme for ranking potential HLA-A2 binding peptides based on independent binding of individual peptide side-chains.. J Immunol.

[19] Doytchinova IA, Guan P, Flower DR (2006). EpiJen: a server for multistep T cell epitope prediction.. BMC Bioinformatics.

[20] Peters B, Bui HH, Frankild S, Nielson M, Lundegaard C (2006). A community resource benchmarking predictions of peptide binding to MHC-I molecules.. PLoS Comput Biol.

[21] Larsen MV, Lundegaard C, Lamberth K, Buus S, Lund O (2007). Large-scale validation of methods for cytotoxic T-lymphocyte epitope prediction.. BMC Bioinformatics.

[22] Larsen MV, Lundegaard C, Lamberth K, Buus S, Brunak S (2005). An integrative approach to CTL epitope prediction: a combined algorithm integrating MHC class I binding, TAP transport effciency, and proteasomal cleavage predictions.. Eur J Immunol.

[23] Tenzer S, Peters B, Bulik S, Schoor O, Lemmel C (2005). Modeling the MHC class I pathway by combining predictions of proteasomal cleavage, TAP transport and MHC class I binding.. Cell Mol Life Sci.

[24] Hakenberg J, Nussbaum AK, Schild H, Rammensee HG, Kuttler C (2003). MAPPP: MHC class I antigenic peptide processing prediction.. Appl Bioinformatics.

[25] Burroughs NJ, de Boer RJ, Keşmir C (2004). Discriminating self from nonself with short peptides from large proteomes.. Immunogenetics.

[26] Lundegaard C, Nielsen M, Lund O (2006). The validity of predicted T-cell epitopes.. Trends Biotechnol.

[27] Schellens IMM, Keşmir C, Miedema F, van Baarle D, Borghans JAM (2008). An unanticipated lack of consensus cytotoxic T lymphocyte epitopes in HIV-1 databases: the contribution of prediction programs.. AIDS.

[28] Pérez CL, Larsen MV, Gustafsson R, Norström MM, Atlas A (2008). Broadly immunogenic HLA class I supertype-restricted elite CTL epitopes recognized in a diverse population infected with different HIV-1 subtypes.. J Immunol.

[29] Fortier MH, Caron E, Hardy MP, Voisin G, Lemieux S (2008). The MHC class I peptide repertoire is molded by the transcriptome.. J Exp Med.

[30] Klein MR, van der Burg SH, Hovenkamp E, Holwerda AM, Drijfhout JW (1998). Characterization of HLA-B57-restricted human immunodeficiency virus type 1 GAG- and RT-specific cytotoxic T lymphocyte responses.. J Gen Virol.

[31] Kaslow RA, Rivers C, Tang J, Bender TJ, Goepfert PA (2001). Polymorphisms in HLA class I genes associated with both favorable prognosis of human immunodeficiency virus (HIV) type 1 infection and positive cytotoxic T-lymphocyte responses to ALVACHIV recombinant canarypox vaccines.. J Virol.

[32] Kuiken C, Korber B, Shafer RW (2003). HIV sequence databases.. AIDS Rev.

[33] Kendall MG (1938). A new measure of rank correlation.. Biometrika.

[34] Ihaka R, Gentleman R (1996). R: A language for data analysis and graphics.. Journal of Computational and Graphical Statistics.

[35] Korber B, Muldoon M, Theiler J, Gao F, Gupta R (2000). Timing the ancestor of the HIV-1 pandemic strains.. Science.

[36] Eddy SR (1998). Profile hidden Markov models.. Bioinformatics.

[37] Slade RW, McCallum HI (1992). Overdominant vs. frequency-dependent selection at MHC loci.. Genetics.

[38] Müller V, Ledergerber B, Perrin L, Klimkait T, Furrer H (2006). Stable virulence levels in the HIV epidemic of Switzerland over two decades.. AIDS.

[39] Herbeck JT, Gottlieb GS, Li X, Hu Z, Detels R (2008). Lack of evidence for changing virulence of HIV-1 in North America.. PLoS ONE.

[40] Moore CB, John M, James IR, Christiansen FT, Witt CS (2002). Evidence of HIV-1 adaptation to HLA-restricted immune responses at a population level.. Science.

[41] Leslie A, Kavanagh D, Honeyborne I, Pfafferott K, Edwards C (2005). Transmission and accumulation of CTL escape variants drive negative associations between HIV polymorphisms and HLA.. J Exp Med.

[42] Poon AFY, Pond SLK, Bennett P, Richman DD, Brown AJL (2007). Adaptation to human populations is revealed by within-host polymorphisms in HIV-1 and hepatitis C virus.. PLoS Pathog.

[43] Bhattacharya T, Daniels M, Heckerman D, Foley B, Frahm N (2007). Founder effects in the assessment of HIV polymorphisms and HLA allele associations.. Science.

[44] Brumme ZL, Brumme CJ, Heckerman D, Korber BT, Daniels M (2007). Evidence of differential HLA class I-mediated viral evolution in functional and accessory/regulatory genes of hiv-1.. PLoS Pathog.

[45] Yusim K, Kesmir C, Gaschen B, Addo MM, Altfeld M (2002). Clustering patterns of cytotoxic T-lymphocyte epitopes in human immunodeficiency virus type 1 (HIV-1) proteins reveal imprints of immune evasion on HIV-1 global variation.. J Virol.

[46] Zhang D, Shankar P, Xu Z, Harnisch B, Chen G (2003). Most antiviral CD8 T cells during chronic viral infection do not express high levels of perforin and are not directly cytotoxic.. Blood.

[47] Asquith B, Edwards CTT, Lipsitch M, McLean AR (2006). Ineffcient cytotoxic T lymphocyte-mediated killing of HIV-1-infected cells in vivo.. PLoS Biol.

[48] Furutsuki T, Hosoya N, Kawana-Tachikawa A, Tomizawa M, Odawara T (2004). Frequent transmission of cytotoxic-T-lymphocyte escape mutants of human immunodeficiency virus type 1 in the highly HLA-A24-positive japanese population.. J Virol.

[49] Li B, Gladden AD, Altfeld M, Kaldor JM, Cooper DA (2007). Rapid reversion of sequence polymorphisms dominates early human immunodeficiency virus type 1 evolution.. J Virol.

[50] Frater AJ, Brown H, Oxenius A, Günthard HF, Hirschel B (2007). Effective T-cell responses select human immunodeficiency virus mutants and slow disease progression.. J Virol.

[51] Matano T, Shibata R, Siemon C, Connors M, Lane HC (1998). Administration of an anti-CD8 monoclonal antibody interferes with the clearance of chimeric simian/human immunodeficiency virus during primary infections of rhesus macaques.. J Virol.

[52] Jin X, Bauer DE, Tuttleton SE, Lewin S, Gettie A (1999). Dramatic rise in plasma viremia after CD8(+) T cell depletion in simian immunodeficiency virus-infected macaques.. J Exp Med.

[53] Schmitz JE, Kuroda MJ, Santra S, Sasseville VG, Simon MA (1999). Control of viremia in simian immunodeficiency virus infection by CD8+ lymphocytes.. Science.

[54] Carrington M, Nelson GW, Martin MP, Kissner T, Vlahov D (1999). HLA and HIV-1: heterozygote advantage and B*35-Cw*04 disadvantage.. Science.

[55] Yewdell JW, Bennink JR (1999). Immunodominance in major histocompatibility complex class I-restricted T lymphocyte responses.. Annu Rev Immunol.

[56] Rolland M, Nickle DC, Mullins JI (2007). HIV-1 group M conserved elements vaccine.. PLoS Pathog.

[57] Frankild S, de Boer RJ, Lund O, Nielsen M, Kesmir C (2008). Amino acid similarity accounts for T cell cross-reactivity and for “holes” in the T cell repertoire.. PLoS ONE.

[58] Zafiropoulos A, Barnes E, Piggott C, Klenerman P (2004). Analysis of ‘driver’ and ‘passenger’ CD8+ T-cell responses against variable viruses.. Proc Biol Sci.

[59] van Baalen CA, Guillon C, van Baalen M, Verschuren EJ, Boers PHM (2002). Impact of antigen expression kinetics on the effectiveness of HIV-specific cytotoxic T lymphocytes.. Eur J Immunol.

[60] Sacha JB, Chung C, Rakasz EG, Spencer SP, Jonas AK (2007). Gag-specific CD8+ T lymphocytes recognize infected cells before AIDS-virus integration and viral protein expression.. J Immunol.

[61] Sacha JB, Chung C, Reed J, Jonas AK, Bean AT (2007). Pol-specific CD8+ T cells recognize simian immunodeficiency virus-infected cells prior to NEF-mediated major histocompatibility complex class I downregulation.. J Virol.

[62] Whitney JB, Cobb RR, Popp RA, O'Rourke TW (1985). Detection of neutral amino acid substitutions in proteins.. Proc Natl Acad Sci U S A.

[63] Kimura M (1991). Recent development of the neutral theory viewed from the wrightian tradition of theoretical population genetics.. Proc Natl Acad Sci U S A.

[64] Yokomaku Y, Miura H, Tomiyama H, Kawana-Tachikawa A, Takiguchi M (2004). Impaired processing and presentation of cytotoxic-T-lymphocyte (CTL) epitopes are major escape mechanisms from CTL immune pressure in human immunodeficiency virus type 1 infection.. J Virol.

[65] Gaschen B, Taylor J, Yusim K, Foley B, Gao F (2002). Diversity considerations in HIV-1 vaccine selection.. Science.

[66] Brander C, Yang OO, Jones NG, Lee Y, Goulder P (1999). Effcient processing of the immunodominant, HLA-A*0201-restricted human immunodeficiency virus type 1 cytotoxic T-lymphocyte epitope despite multiple variations in the epitope flanking sequences.. J Virol.

[67] Allen TM, Yu XG, Kalife ET, Reyor LL, Lichterfeld M (2005). De novo generation of escape variant-specific CD8+ t-cell responses following cytotoxic t-lymphocyte escape in chronic human immunodeficiency virus type 1 infection.. J Virol.

[68] Karlsson AC, Iversen AKN, Chapman JM, de Oliviera T, Spotts G (2007). Sequential broadening of CTL responses in early HIV-1 infection is associated with viral escape.. PLoS ONE.

[69] Barouch DH, Powers J, Truitt DM, Kishko MG, Arthur JC (2005). Dynamic immune responses maintain cytotoxic T lymphocyte epitope mutations in transmitted simian immunodeficiency virus variants.. Nat Immunol.

[70] Gubler B, Daniel S, Armandola EA, Hammer J, Caillat-Zucman S (1998). Substrate selection by transporters associated with antigen processing occurs during peptide binding to TAP.. Mol Immunol.

[71] Walker BA, van Hateren A, Milne S, Beck S, Kaufman J (2005). Chicken TAP genes differ from their human orthologues in locus organisation, size, sequence features and polymorphism.. Immunogenetics.

[72] Navis M, Schellens I, van Baarle D, Borghans J, van Swieten P (2007). Viral replication capacity as a correlate of HLA B57/B5801-associated nonprogressive HIV-1 infection.. J Immunol.

[73] Kutsch O, Vey T, Kerkau T, Hünig T, Schimpl A (2002). HIV type 1 abrogates TAP-mediated transport of antigenic peptides presented by MHC class I. transporter associated with antigen presentation.. AIDS Res Hum Retroviruses.

[74] Baugh LL, Garcia JV, Foster JL (2008). Functional characterization of the human immunodeficiency virus type 1 NEF acidic domain.. J Virol.

[75] Maurer K, Harrer EG, Goldwich A, Eismann K, Bergmann S (2008). Role of cytotoxic T-lymphocyte-mediated immune selection in a dominant human leukocyte antigen-B8-restricted cytotoxic T-lymphocyte epitope in NEF.. J Acquir Immune Defic Syndr.

