# Correction: The Specificity and Polymorphism of the MHC Class I Prevents the Global Adaptation of HIV-1 to the Monomorphic Proteasome and TAP

**DOI:** 10.1371/annotation/98c3dca8-871e-475e-aaed-c10af4dcf35c

**Published:** 2009-01-26

**Authors:** Boris V. Schmid, Can Keşmir, Rob J. de Boer

The first author's name appears incorrectly. It should read: Boris V. Schmid. The correct citation should read: Schmid BV, Keşmir C, de Boer RJ (2008) The Specificity and Polymorphism of the MHC Class I Prevents the Global Adaptation of HIV-1 to the Monomorphic Proteasome and TAP. PLoS ONE 3(10): e3525. doi:10.1371/journal.pone.0003525

