# Supplementary material for: The Specificity and Polymorphism of the MHC Class I Prevents the Global Adaptation of HIV-1 to the Monomorphic Proteasome and TAP
Source: PLoS One. 2008 Oct 24;3(10):e3525. doi: 10.1371/journal.pone.0003525 (PMC2569417; doi:10.1371/journal.pone.0003525)
Supplement: Table S1 — (0.03 MB DOC) [file pone.0003525.s001.doc]

Table S1: Details longitudinal within-host data set (part 1)

| **Patient ID** | **Protein** | **Sampling date** | **Accession number(s)** |
| --- | --- | --- | --- |
|  | | | |
| 005(10151393) | NEF | 1985 | AF129336 |
|  |  | 1989 | AF129395 |
| PIC1362(10152829) | GAG | 1998 | DQ853466, DQ853467, DQ853468, DQ853469, DQ853470, DQ853471, DQ853472, DQ853473, DQ853475 |
|  |  | 2002 | DQ853439, DQ853440, DQ853441, DQ853442, DQ853443, DQ853444, DQ853445, DQ853446, DQ853447, DQ853448 |
|  | NEF | 1998 | DQ853427, DQ853430, DQ853432, DQ853605, DQ853607, DQ853608, DQ853611, DQ853612, DQ853614, DQ853615, DQ853616, DQ853618, DQ853620, DQ853622, DQ853624, DQ853625, DQ853626, DQ853627, DQ853628, DQ853629,  DQ853630, DQ853632, DQ853633, DQ853634, DQ853635, DQ853636, DQ853637, DQ853642, DQ853645, DQ853646, DQ853647, DQ853648 |
|  |  | 2002 | DQ853439, DQ853440, DQ853441, DQ853442, DQ853443, DQ853444, DQ853445, DQ853446, DQ853448, DQ853486, DQ853487, DQ853488, DQ853489, DQ853490, DQ853491, DQ853492, DQ853493, DQ853494, DQ853495, DQ853496, DQ853497, DQ853498, DQ853499, DQ853500 |
|  | POL | 1998 | DQ853466, DQ853467, DQ853468, DQ853469, DQ853470, DQ853471, DQ853472, DQ853473, DQ853474 |
|  |  | 2002 | DQ853439, DQ853440, DQ853441, DQ853442, DQ853443, DQ853444, DQ853445, DQ853446, DQ853447, DQ853448 |

For patient PIC1362 additional sequence information is available in the Los Alamos Database for ENV, REV, TAT, VIF, VPR and VPU. To have at least more than one patient per protein we have limited our analysis of the available sequence data of patient PIC1362 to GAG, NEF and POL.
