# Supplementary material for: The Specificity and Polymorphism of the MHC Class I Prevents the Global Adaptation of HIV-1 to the Monomorphic Proteasome and TAP
Source: PLoS One. 2008 Oct 24;3(10):e3525. doi: 10.1371/journal.pone.0003525 (PMC2569417; doi:10.1371/journal.pone.0003525)
Supplement: Table S2 — (0.05 MB DOC) [file pone.0003525.s002.doc]

Table S2: Details longitudinal within-host data set (part 2)

| **Patient ID** | **Protein** | **Sampling date** | **Accession number** |
| --- | --- | --- | --- |
|  | | | |
| MACS1(10159224) | GAG | 1985 | EF525480 |
|  |  | 1989 | EF525482 |
|  | POL | 1985 | EF525481 |
|  |  | 1989 | EF525483 |
| MACS2(10159231) | GAG | 1991 | EF525500 |
|  |  | 1995 | EF525502 |
|  | POL | 1991 | EF525501 |
|  |  | 1995 | EF525503 |
| MACS3(10159232) | GAG | 1987 | EF525504 |
|  |  | 1992 | EF525505 |
| MACS5(10159234) | GAG | 1984 | EF525512 |
|  |  | 1989 | EF525514 |
|  | POL | 1984 | EF525513 |
|  |  | 1989 | EF525515 |
| MACS7(10159236) | GAG | 1988 | EF525520 |
|  |  | 1992 | EF525522 |
|  | POL | 1988 | EF525521 |
|  |  | 1992 | EF525523 |
| MACS9(10159238) | GAG | 1992 | EF525526 |
|  |  | 1997 | EF525528 |
|  | POL | 1992 | EF525527 |
|  |  | 1997 | EF525529 |
| MACS11(10159226) | GAG | 1985 | EF525488 |
|  |  | 1990 | EF525490 |
|  | POL | 1985 | EF525489 |
|  |  | 1990 | EF525491 |
| MACS12(10159227) | GAG | 1985 | EF525492 |
|  |  | 1992 | EF525493 |
| MACS13(10159228) | POL | 1984 | EF525494 |
|  |  | 1989 | EF525495 |
| MACS14(10159229) | POL | 1986 | EF525496 |
|  |  | 1988 | EF525497 |
| MACS16(10159230) | POL | 1989 | EF525498 |
|  |  | 1994 | EF525499 |
