# Supplementary material for: The Specificity and Polymorphism of the MHC Class I Prevents the Global Adaptation of HIV-1 to the Monomorphic Proteasome and TAP
Source: PLoS One. 2008 Oct 24;3(10):e3525. doi: 10.1371/journal.pone.0003525 (PMC2569417; doi:10.1371/journal.pone.0003525)
Supplement: Table S3 — (0.05 MB DOC) [file pone.0003525.s003.doc]

Table S3: Details HIV-1 Clade B population data set

| *Protein (# samples)* |  | *P* | *density per aa* | *2008->2032* | *half-life* |
| --- | --- | --- | --- | --- | --- |
|  | | | | | |
| ENV (196) |  | | | | |
|  | Precursors | 0.6552 | 0.319 | 273.1->272.0 | 3083 y |
|  | MHC-binders | **0.0085** | 0.037 | 31.3->31.8 |  |
|  | Epitopes | 0.2145 | 0.019 | 16.0->16.3 |  |
| GAG (186) |  | | | | |
|  | Precursors | 0.9887 | 0.237 | 117.4->117.6 |  |
|  | MHC-binders | 0.4551 | 0.029 | 14.5->14.4 | 2924 y |
|  | Epitopes | 0.5673 | 0.010 | 4.9->4.9 | 1033 y |
| NEF (368) |  | | | | |
|  | Precursors | 0.0188 | 0.266 | 55.2->56.6 |  |
|  | MHC-binders | 0.6518 | 0.030 | 6.2->6.2 | 83727 y |
|  | Epitopes | 0.0231 | 0.014 | 2.9->3.0 |  |
| POL (97) |  | | | | |
|  | Precursors | 0.0825 | 0.257 | 257.4->254.4 | 1042 y |
|  | MHC-binders | 0.6235 | 0.033 | 32.6->32.5 | 4384 y |
|  | Epitopes | 0.0424 | 0.013 | 12.6->12.3 | 526 y |
| REV (96) |  | | | | |
|  | Precursors | 0.3933 | 0.216 | 25.5->25.1 | 858 y |
|  | MHC-binders | 0.3137 | 0.027 | 3.2->3.1 | 359 y |
|  | Epitopes | 0.1570 | 0.009 | 1.0->0.9 | 127 y |
| TAT (99) |  | | | | |
|  | Precursors | 0.9730 | 0.211 | 18.3->18.4 |  |
|  | MHC-binders | 0.7891 | 0.017 | 1.5->1.5 |  |
|  | Epitopes | 0.2038 | 0.004 | 0.3->0.4 |  |
| VIF (180) |  | | | | |
|  | Precursors | 0.0388 | 0.271 | 52.1->53.8 |  |
|  | MHC-binders | **0.0037** | 0.037 | 7.1->6.9 | 428 y |
|  | Epitopes | 0.3610 | 0.013 | 2.6->2.6 | 1986 y |
| VPR (160) |  | | | | |
|  | Precursors | 0.1176 | 0.326 | 31.3->30.4 | 444 y |
|  | MHC-binders | 0.0206 | 0.035 | 3.4->3.3 | 352 y |
|  | Epitopes | **0.0037** | 0.016 | 1.5->1.4 | 124 y |
| VPU (147) |  | | | | |
|  | Precursors | 0.2528 | 0.337 | 27.7->28.7 |  |
|  | MHC-binders | 0.5678 | 0.052 | 4.3->4.2 | 1426 y |
|  | Epitopes | 0.3480 | 0.025 | 2.1->2.0 | 464 y |

The density of epitope precursors, MHC-binding 9mers and CTL epitopes is expressed per amino-acid and where applicable averaged over the 32 available MHC-binding predictors. The ‘2008->32’ column gives the estimated current (2008) number of precursors, average number of MHC-binders and average number of CTL epitopes and projects 25 years into the future, based on linear regression. ‘Half-life’ is an estimate of the number of years it will take at which half of the precursors, MHC-binders or CTL epitopes have been lost, assuming a linear decline. Statistical test: Kendall Tau rank correlation test, with p-values < 0.001 in bold face.
