# Supplementary material for: The Specificity and Polymorphism of the MHC Class I Prevents the Global Adaptation of HIV-1 to the Monomorphic Proteasome and TAP
Source: PLoS One. 2008 Oct 24;3(10):e3525. doi: 10.1371/journal.pone.0003525 (PMC2569417; doi:10.1371/journal.pone.0003525)
Supplement: Table S4 — (0.05 MB DOC) [file pone.0003525.s004.doc]

Table S4: Details HIV-1 Clade C population data set

| *Protein (# samples)* |  | *P* | *density per aa* | *2008->2032* | *half-life* |
| --- | --- | --- | --- | --- | --- |
|  | | | | | |
| ENV (346) |  | | | | |
|  | Precursors | 0.8204 | 0.319 | 273.5->271.9 | 2108 y |
|  | MHC-binders | 0.0933 | 0.037 | 31.3->31.2 | 3580 y |
|  | Epitopes | 0.2452 | 0.019 | 15.9->15.7 | 978 y |
| GAG (577) |  | | | | |
|  | Precursors | 0.2472 | 0.239 | 118.3->116.8 | 914 y |
|  | MHC-binders | 0.0357 | 0.030 | 14.6->14.5 | 2222 y |
|  | Epitopes | **0.0003** | 0.009 | 4.6->4.3 | 150 y |
| NEF (374) |  | | | | |
|  | Precursors | **0.0007** | 0.269 | 55.7->51.5 | 160 y |
|  | MHC-binders | 0.1038 | 0.030 | 6.2->5.9 | 227 y |
|  | Epitopes | 0.0152 | 0.015 | 3.1->2.8 | 121 y |
| POL (279) |  | | | | |
|  | Precursors | 0.7939 | 0.257 | 257.9->256.7 | 2582 y |
|  | MHC-binders | 0.9384 | 0.032 | 32.5->32.5 | 180095 y |
|  | Epitopes | 0.5383 | 0.012 | 12.4->12.5 |  |
| REV (295) |  | | | | |
|  | Precursors | 0.1672 | 0.193 | 22.7->21.2 | 181 y |
|  | MHC-binders | 0.7996 | 0.027 | 3.2->3.2 | 18684 y |
|  | Epitopes | 0.6159 | 0.006 | 0.8->0.7 | 171 y |
| TAT (286) |  | | | | |
|  | Precursors | 0.4717 | 0.187 | 16.3->16.6 |  |
|  | MHC-binders | **0.0059** | 0.017 | 1.5->1.3 | 81 y |
|  | Epitopes | 0.4379 | 0.004 | 0.3->0.3 | 649 y |
| VIF (295) |  | | | | |
|  | Precursors | 0.6219 | 0.286 | 54.9->55.0 |  |
|  | MHC-binders | 0.0593 | 0.037 | 7.1->7.4 |  |
|  | Epitopes | 0.6649 | 0.013 | 2.6->2.5 | 1053 y |
| VPR (298) |  | | | | |
|  | Precursors | 0.9737 | 0.313 | 30.1->30.0 | 8964 y |
|  | MHC-binders | 0.2668 | 0.035 | 3.3->3.3 | 656 y |
|  | Epitopes | 0.4666 | 0.016 | 1.5->1.6 |  |
| VPU (293) |  | | | | |
|  | Precursors | 0.8715 | 0.376 | 30.8->30.8 |  |
|  | MHC-binders | 0.6532 | 0.053 | 4.3->4.2 | 461 y |
|  | Epitopes | 0.4382 | 0.023 | 1.9->1.8 | 223 y |

See Table S3 for an explanation of the columns. Removed proteins with less than 50 samples. Statistical test: Kendall Tau rank correlation test, with p-values < 0.001 in bold face.
