# Supplementary material for: The Specificity and Polymorphism of the MHC Class I Prevents the Global Adaptation of HIV-1 to the Monomorphic Proteasome and TAP
Source: PLoS One. 2008 Oct 24;3(10):e3525. doi: 10.1371/journal.pone.0003525 (PMC2569417; doi:10.1371/journal.pone.0003525)
Supplement: Table S5 — (0.05 MB DOC) [file pone.0003525.s005.doc]

Table S5: Details HIV-1 Clade A1 population data set

| *Protein (# samples)* |  | *P* | *density per aa* | *2008->2032* | *half-life* |
| --- | --- | --- | --- | --- | --- |
|  | | | | | |
| ENV (51) |  | | | | |
|  | Precursors | **0.0002** | 0.303 | 259.1->240.7 | 169 y |
|  | MHC-binders | 0.4856 | 0.036 | 30.8->30.4 | 951 y |
|  | Epitopes | 0.3581 | 0.018 | 15.5->15.1 | 470 y |
| GAG (118) |  | | | | |
|  | Precursors | 0.9002 | 0.233 | 115.5->117.2 |  |
|  | MHC-binders | **0.0026** | 0.030 | 14.7->15.4 |  |
|  | Epitopes | 0.0156 | 0.010 | 4.7->5.1 |  |
| NEF (87) |  | | | | |
|  | Precursors | 0.5081 | 0.254 | 52.6->50.4 | 291 y |
|  | MHC-binders | 0.3472 | 0.030 | 6.3->6.3 |  |
|  | Epitopes | 0.6436 | 0.015 | 3.0->3.0 |  |
| POL (54) |  | | | | |
|  | Precursors | 0.1073 | 0.254 | 254.1->257.0 |  |
|  | MHC-binders | 0.0852 | 0.033 | 33.0->33.3 |  |
|  | Epitopes | 0.0242 | 0.012 | 12.2->12.5 |  |
| REV (58) |  | | | | |
|  | Precursors | 0.4442 | 0.183 | 21.6->20.3 | 195 y |
|  | MHC-binders | 0.3422 | 0.024 | 2.9->2.9 | 2656 y |
|  | Epitopes | 0.8804 | 0.005 | 0.6->0.7 |  |
| TAT (55) |  | | | | |
|  | Precursors | 0.9132 | 0.191 | 16.6->15.7 | 212 y |
|  | MHC-binders | 0.9172 | 0.018 | 1.6->1.6 | 440 y |
|  | Epitopes | **0.0062** | 0.003 | 0.3->-0.0 | 11 y |
| VIF (62) |  | | | | |
|  | Precursors | 0.0213 | 0.265 | 50.8->47.8 | 202 y |
|  | MHC-binders | 0.1021 | 0.035 | 6.7->6.9 |  |
|  | Epitopes | 0.7905 | 0.013 | 2.4->2.5 |  |
| VPR (59) |  | | | | |
|  | Precursors | 0.1106 | 0.294 | 28.2->29.2 |  |
|  | MHC-binders | 0.1236 | 0.033 | 3.1->3.0 | 257 y |
|  | Epitopes | 0.8780 | 0.015 | 1.5->1.5 |  |
| VPU (66) |  | | | | |
|  | Precursors | 0.1275 | 0.342 | 28.0->28.4 |  |
|  | MHC-binders | **0.0046** | 0.050 | 4.1->4.8 |  |
|  | Epitopes | **0.0011** | 0.025 | 2.0->2.5 |  |

See Table S3 for an explanation of the columns. Removed proteins with less than 50 samples. Statistical test: Kendall Tau rank correlation test, with p-values < 0.001 in bold face.
