# Supplementary material for: The Specificity and Polymorphism of the MHC Class I Prevents the Global Adaptation of HIV-1 to the Monomorphic Proteasome and TAP
Source: PLoS One. 2008 Oct 24;3(10):e3525. doi: 10.1371/journal.pone.0003525 (PMC2569417; doi:10.1371/journal.pone.0003525)
Supplement: Table S6 — (0.05 MB DOC) [file pone.0003525.s006.doc]

Table S6: Details HIV-1 Clade B sub-population data set

| *Protein (# samples)* |  | *P* | *density per aa* | *2008->2032* | *half-life* |
| --- | --- | --- | --- | --- | --- |
|  | | | | | |
| **HIV-1 Clade B Kroatia** | | | | | |
| NEF (153) |  | | | | |
|  | Precursors | **0.0033** | 0.272 | 56.2->59.2 |  |
|  | MHC-binders | 0.8802 | 0.030 | 6.2->6.3 |  |
|  | Epitopes | **0.0090** | 0.014 | 3.0->3.2 |  |
| VIF (70) |  | | | | |
|  | Precursors | 0.3064 | 0.271 | 52.0->53.6 |  |
|  | MHC-binders | 0.0749 | 0.036 | 7.0->6.7 | 292 y |
|  | Epitopes | 0.1858 | 0.013 | 2.5->2.4 | 203 y |
|  | | | | | |
| **HIV-1 Clade B Great Brittain** | | | | | |
| NEF (60) |  | | | | |
|  | Precursors | 0.6408 | 0.257 | 53.1->52.4 | 854 y |
|  | MHC-binders | 0.5277 | 0.029 | 6.1->5.7 | 169 y |
|  | Epitopes | 0.7420 | 0.013 | 2.8->2.6 | 177 y |
|  | | | | | |
| **HIV-1 Clade B USA** | | | | | |
| ENV (81) |  | | | | |
|  | Precursors | 0.1717 | 0.321 | 275.1->277.2 |  |
|  | MHC-binders | **0.0079** | 0.037 | 31.7->32.6 |  |
|  | Epitopes | 0.2258 | 0.019 | 16.1->16.7 |  |
| GAG (56) |  | | | | |
|  | Precursors | 0.5596 | 0.236 | 116.9->116.0 | 1587 y |
|  | MHC-binders | 0.9887 | 0.029 | 14.5->14.5 | 4273 y |
|  | Epitopes | 0.3232 | 0.010 | 4.8->4.6 | 248 y |
| NEF (62) |  | | | | |
|  | Precursors | 0.5100 | 0.270 | 56.0->57.3 |  |
|  | MHC-binders | 0.3156 | 0.031 | 6.4->6.7 |  |
|  | Epitopes | 0.9902 | 0.014 | 2.9->2.9 |  |
| VPU (56) |  | | | | |
|  | Precursors | 0.4164 | 0.329 | 27.0->26.7 | 1335 y |
|  | MHC-binders | 0.4952 | 0.051 | 4.2->4.1 | 490 y |
|  | Epitopes | 0.3067 | 0.025 | 2.1->1.9 | 151 y |

See Table S3 for an explanation of the columns. Removed proteins with less than 50 samples. Statistical test: Kendall Tau rank correlation test, with p-values < 0.001 in bold face.
